# The Association between *TNF-alpha* Gene Polymorphisms and Endometriosis in An Iranian Population

**DOI:** 10.22074/ijfs.2019.5542

**Published:** 2019-01-06

**Authors:** Babak Babaabasi, Ali Ahani, Faegheh Sadeghi, Haniyeh Bashizade-Fakhar, Hamid Reza Khorram Khorshid

**Affiliations:** 1 Department of Genetics, Reproductive Biomedicine Research Center, Royan Institute for Reproductive Biomedicine, ACECR, Tehran, Iran; 2 Reproductive Biotechnology Research Center, Avicenna Research Institute, ACECR, Tehran, Iran; 3 Mendel Medical Genetics Laboratory, Tehran, Iran; 4 Department of Molecular Biology, Ahar Branch, Islamic Azad University, Ahar, Iran; 5 Department of Laboratory Science, Chalous Branch, Islamic Azad University, Chalous, Iran; 6 Genetic Research Center, University of Social Welfare and Rehabilitation Sciences, Tehran, Iran

**Keywords:** Body Mass Index, Endometriosis, Polymorphisms, Restriction Fragment Length Polymorphism, *TNF-α*

## Abstract

**Background:**

Tumor necrosis factor-alpha (*TNF-α*) is an important cytokine in acute inflammatory response to infective factors. Based on investigation in different populations, it is thought that this response increases in patients with endometrio- sis due to the presence of cytokines such as *TNF-α*. This study aimed to examine the association of four *TNF-α* polymor- phisms, namely -238G/A, -308G/A, -857C/T and -863C/A, with susceptibility to endometriosis in an Iranian population.

**Materials and Methods:**

We recruited 150 women with endometriosis and 150 women without endometriosis in this case-control study and collected 4 ml of blood from all subjects. After DNA extraction, the polymorphisms were geno- typed by polymerase chain reaction-restriction fragment length polymorphism (PCR-RFLP).

**Results:**

The allele frequency of *TNF-α* -863C/A in the case and control groups showed a significant difference [odds ratios (OR)=0.64, 95% confidence interval (CI)=0.41-0.99, P=0.047] but the result is not significant when Adjust- ing for multiple testing (P=0.188). No significant difference in the allele frequencies of -238G/A (OR=1.07, 95% CI=0.51-2.25, P=0.862), -308G/A (OR=0.79, 95% CI=0.43-1.45, P=0.438) and -857C/T (OR=1.03, 95% CI=0.66- 1.61, P=0.887) was observed. We adjusted all four polymorphism genotypes by age and body mass index (BMI), however, no significant difference was detected. There was an association between the case and control and BMI when adjusting by age (OR=1.082, 95% CI=1.009-1.162, P=0.028).

**Conclusion:**

For the first time the association of the four polymorphisms in the promoter region of the *TNF-α* gene with endometriosis has been conducted in women of Iranian origin. The present research reveals the -863 A allele may play a role in incidence of endometriosis among Iranian women. Development of endometriosis among those people with -863 A allele seems low. According to the results, the current study indicates that there might be a correlation between BMI and progression of endometriosis.

## Introduction

Endometriosis is developed as a result of endometrial tissue exposing outside the uterine cavity. Studies have reported the pelvic and the peritoneum as the most common sites of replacement (1, 2). This highly prevalent disease can be really enervating (about 30% in infertile and 10% in fertile women) (3). Approximately, 25-50% of infertile women develop endometriosis while 30-50% of women with endometriosis are infertile (4).

This polygenic disease with its complex genetic background (5, 6) occurs as a result of interactions between genetically determined factors and environment. The genetic component of endometriosis has been shown through studying the kinship of patients (7, 8). To date, the most common method for investigating genetic factors underlying complex diseases is the hypothesis-based candidate gene studies (8). One of the most important factors in endometriosis is mutations in cytokine genes. The tumor necrosis factor-alpha (*TNF-α*) is an important cytokine in acute inflammatory response to infective factors and is genetically variable. Based on multiple studies, *TNF-α* is thought to be a molecular indicator for gynecological-related diseases. It is suggested that the inflammatory response in endometriosis increases because of cytokines such as *TNF-α* (8, 9).

Studies on patients diagnosed with endometriosis have highlighted that *TNF-α* is a likely factor in developing endometriosis as suggested by elevated levels of *TNF-α* in peritoneal fluid and the up-regulation of *TNF-α* in peritoneal macrophages and peripheral blood monocytes (10, 11). However, the exact role which *TNF-α* plays in endometrial tissue is ambiguous (12). Thus far, some polymorphisms in the promoter region of the *TNF-α* gene have been examined in patients with endometriosis (12-20). Therefore, for the first time, we aimed to examine the relationship of *TNF-α* -238G/A, -308G/A, -857C/T and -863C/A polymorphisms with risk of developing endometriosis in Iranian women.

## Materials and Methods

### Subjects

This case-control study enrolled a total of 150 Iranian women with endometriosis who had referred to Avicenna Infertility Clinic and Tehran Clinic Hospital, Tehran, Iran. Diagnostic laparoscopy was performed in all patients. The severity of endometriosis was determined using the revised American Society for Reproductive Medicine (ASRM) classification (stages I-IV of disease). The control group consisted of 150 women without endometriosis. Only women who underwent laparoscopy for non-endometriosis infertility and showed absence of endometriosis were included as controls. Stages I and II of endometriosis are commonly found in asymptomatic women (21). The exclusion criteria in our study were the following: having a history of rheumatoid arthritis, diabetic retinopathy and Behcet’s disease. Approval from the Avicenna Research Institute Ethics and Human Rights Committee was obtained for using blood samples and the designed protocol. Written informed consent was obtained from all patients with inclusion criteria to take part in the study.

### DNA extraction and genotyping

Blood was collected in tubes with 200 µl EDTA (0.5 M), as an anti-clotting factor, and stored at -20ºC until DNA extraction. Genomic DNA was extracted by salting out method from peripheral blood samples. Genotyping of the -238G/A (rs361525), -308G/A (rs1800629), -857C/ T (rs1799724) and -863C/A (rs1800630) polymorphisms in the 5'-untranslated region of *TNF-α* was performed using polymerase chain reaction-restriction fragment length polymorphism (PCR-RFLP). Details on primers and restriction enzymes are presented in Table 1.

The PCR reactions carried out in final volume of 25 µl containing: 10X PCR Buffer (Roche, Germany), 1.5 mM MgCl_2_ (Roche, Germany), 0.4 µM of each dNTP (Fermentas, Germany), 5 pmol of each primer, 50 ng template DNA, 1 U Taq DNA polymerase (Roche, Germany) and sterile distilled water up to 25 µl. Amplification conditions start with an initial denaturation step of 5 minutes at 94ºC, followed by 30 cycles of 30 seconds denaturation (94ºC), 30 seconds annealing (63ºC) for -238G/A, -857C/T and -863C/A and 30 seconds annealing (66ºC) for -308G/A and 30 seconds extension (72ºC), ended by a final extension for 5 minutes (72ºC) and finally cooling to 4ºC.

**Fig.1:**
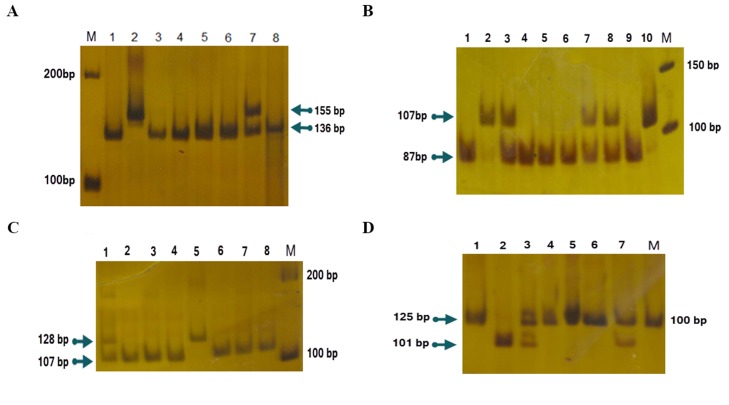
Representative gel pictures of polymerase chain reaction-restriction fragment length polymorphism (PCR-RFLP) results. **A.** The -238G/A polymorphism PCR-RFLP result. Lane M; Ladder 100 bp, No. 1, 3-6 and 8; Homozygote (GG), No. 2; Homozygote (AA), No. 7; Heterozygote (GA), **B.** The -308G/A polymorphism PCR-RFLP result. Lane M; Ladder 50 bp, No.1, 4-6 and 9; Homozygote (GG), No. 3, 7 and 8; Heterozygote (GA), No.2; Homozygote (AA), and No. 10; Undigested PCR product as the control, **C.** The -857C/T polymorphism PCR-RFLP result. Lane M; Ladder 100 bp, No. 2-4 and 6-8; Homozygote (CC), No. 1; Heterozygote (TC) and No. 5; Homozygote (TT), and **D.** The -863C/A polymorphism PCR-RFLP result. Lane M; Ladder 100 bp, No.1 and 2; Heterozygote (AC) and No. 3-5; Homozygote (CC).

Polymerase chain reaction products were electrophoresed on a 1.5% agarose gel in 1X TAE and stained with ethidium bromide and visualized by ultraviolet light. After reviewing the PCR products, they were treated with restriction enzymes (Hpa II and NcoI at 37ºC and TaiI at 65ºC) overnight. The digestion products were subjected to 10% polyacrylamide gel electrophoresis and stained with silver nitrate (Fig .1).

### Statistical analyses

Results were analyzed by SPSS 24.0 software (IBM SPSS Statistical Software, USA). The analysis of age and body mass index (BMI) in the study groups were performed using t test. The allele frequencies were compared using the Chi-squared test. Genotype distributions in the case and control groups were also analyzed. Age and BMI were considered as potential confounders. The analyses were performed and adjusted in terms of age and BMI using logistic regression. P<0.05 was considered statistically significant. The P value corrected using Bonferroni method for the multiple testing. Logistic regression was used to predict the odds of developing a given disease based on observed characteristics of the patients. In our study, the criterion variable was the logistic regression of disease and no-disease. To perform the statistical analysis using SPSS, we considered the two case and control groups as dependent variables. Age and BMI were considered as covariants and genotype selected as the basis of categorical covariant. 

**Table 1:** Information about primers and restriction enzymes used


Gene	Variation	Primers (5ˊ-3ˊ)	Size (bp)	Restriction enzyme	Allele	Cutting product (bp)

*TNF-α*	-238G/A	F: AGAAGACCCCCCTCGGAACC	165	Hpa II (New England BioLabs)	G	136
		R: CTCATCTGGAGGAAGCGGTA				19
	-308G/A	F: AGGCAATAGGTTTTGAGGGCCAT	107	NcoI (New England BioLabs)	G	87
		R: TCCTCCCTGCTCCGATTCCG				20
	-857C/T	F: GGCTCTGAGGAATGGGTTAC	128	TaiI (New England BioLabs)	C	107
		R: CCTCTACATGGCCCTGTCTAC				21
	-863C/A	F: GGCTCTGAGGAATGGGTTAC	125	TaiI (New England BioLabs)	A	101
		R: CTACATGGCCCTGTCTTCGTTACG				24


For interaction analysis, the STRING online server (http://string-db.org/) was used to acquire the network of protein-protein interactions for *TNF-α*.

## Results

According to the analysis of descriptive variables, the age range was from 19 to 50 years (mean=31, SD=6.1) in the patients, and from 19 to 44 years (mean=29.2, SD=5.2) in the control group. The mean BMI (Kg/m^2^) in the case and control groups were 25.2 (SD=3.7) and 26.2 (SD=4) respectively. Genotypes of the *TNF-α* -238G/A, -308G/A, -857C/T and -863C/A polymorphisms were obtained in 150, 150, 148, 150 patients and 149, 150, 143, 150 control samples respectively. Genotype frequencies of the *TNF-α* -238G/A, -308G/A, -857C/T and -863C/A polymorphisms in the case and control groups were in Hardy-Weinberg equilibrium. Genotype and allele frequencies for the *TNF-α* -238G/A, -308G/A, -857C/T and -863C/A are shown in Table 2.

The *TNF-α* -863C/A allele A frequency between case and control groups represented a significant difference (P=0.047) but the result is not significant when adjusting for multiple testing (P=0.188). However, no significant difference was observed in the allele frequencies of the -238G/A (P=0.862), -308G/A (P=0.438) and -857C/T (P=0.878) polymorphisms in *TNF-α* between the case and control groups. We adjusted all four polymorphism genotypes by age and BMI but according to the results, no significant difference was discovered between the groups. But there was an association between the case and control and BMI when adjusting by age (OR=1.082, 95% CI=1.009-1.162, P=0.028).

**Fig.2:**
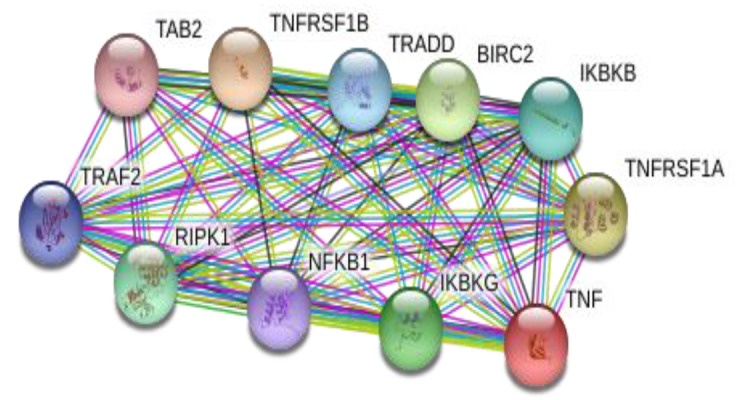
*TNF-α* protein-protein interactions network obtained from STRING (htpp://string-db.org/).

*TNF-α* interacts with 10 other proteins according to STRING (Fig .2). In specific, they are i. TGF-beta activated kinase 1/MAP3K7 binding protein 2 (*TAB2*), ii. Nuclear factor of kappa light polypeptide gene enhancer in B-cells 1 (*NFKB1*), iii. *TNF* receptor-associated factor 2 (*TRAF2*), iv. TNFRSF1A-associated via death domain (TRADD), v. Inhibitor of kappa light polypeptide gene enhancer in B-cells, kinase beta (*IKBKB*), vi. Receptor interacting serine-threonine kinase 1 (RIPK1), vii. Inhibitor of kappa light polypeptide gene enhancer in B-cells, kinase gamma (IKBKG), viii. Baculoviral IAP repeat containing 2 (BIRC2), ix. Tumor necrosis factor receptor superfamily, member 1A (*TNFRSF1A*), and x. Tumor necrosis factor receptor superfamily, member 1B; (*TNFRSF1B*).

**Table 2:** Genotype and allele frequencies of the four polymorphisms in the promoter region of TNF-α in patients with stage I-IV of endometriosis and controls


Polymorphisms^*^		Cases	Controls	OR	95% CI	P value	Corrected P value^**^

The -238 (rs361525)	Genotype	GG	137 (91.3%)	135 (90.6%)				
		GA	11 (7.3%)	14 (9.4%)	0.65	0.24-1.71	0.381	0.762
		AA	2 (1.3%)	0 (0.0%)	NA	NA	NA	NA
	Allele	G	285 (95.0%)	284 (95.3%)				
		A	15 (5.0%)	14 (4.7%)	1.07	0.51-2.25	0.862	1
The -308 (rs1800629)	Genotype	GG	131 (87.3%)	127 (84.7%)				
		GA	18 (12.0%)	21 (14.0%)	0.8	0.35-1.84	0.604	1
		AA	1 (0.7%)	2 (1.3%)	0	NA	0.999	1
	Allele	G	280 (93.3%)	275 (91.7%)				
		A	20 (6.67%)	25 (8.3%)	0.79	0.43-1.45	0.438	1
The -857 (rs1799724)	Genotype	CC	102 (68.9%)	102 (71.3%)				
		CT	43 (29.1%)	36 (25.2%)	1.62	0.86-3.04	0.137	0.274
		TT	3 (2.0%)	5 (3.5%)	0.46	0.05-4.35	0.499	0.998
	Allele	C	247 (83.5%)	240 (83.9%)				
		T	49 (16.5%)	46 (16.1%)	1.03	0.66-1.61	0.887	1
The -863 (rs1800630)	Genotype	CC	114 (76.0%)	99 (66.0%)				
		CA	32 (21.3%)	44 (29.3%)	0.66	0.35-1.27	0.215	0.43
		AA	4 (2.7%)	7 (4.7%)	0.68	0.19-2.47	0.557	1
	Allele	C	260 (86.7%)	242 (80.67%)				
		A	40 (13.3%)	58 (19.3%)	0.64	0.41-0.99	0.047	0.188


OR; Odds ratios, CI; Confidence interval, BMI; Body mass index, NA; No answer, *; The effect of genotypes were adjusted by age and BMI, and **; The P value corrected using Bonferroni method for the multiple testing.

**Table 3:** TNF-α promoter polymorphisms studies


SNP name	Association with susceptibility	
	Association Population/(number of cases and controls)	No-association Population/(number of cases and controls)

-1031T/C	Japanese (123, 165) (Teramoto et al.) (20)Japanese (130, 185) (Asghar et al.) (12)Iranian (135, 173) (Saliminejad et al.) (15)Iranian (65, 65) (Abutorabi et al.) (16)	Australian (958, 959) (Zhao et al.) (19)
-863C/A	Japanese (123, 165) (Teramoto et al.) (20)Iranian (150, 150) (This study)^*, £^	Japanese (130, 185) (Asghar et al.) (12)
-857C/T	Japanese (123, 165) (Teramoto et al.) (20)Iranian (148, 143) (This study)^£^	Japanese (130, 185) (Asghar et al.) (12)Australian (958, 959) (Zhao et al.) (19)Iranian (148, 143) (This study)^**^
-308G/A	Iranian (150, 150) (This study)^£^	Taiwanese (120, 106) (Hsieh et al.) (13)Korean (70, 202) (Lee et al.) (17)Austrian (92, 69) (Wieser et al.) (18)Japanese (130, 185) (Asghar et al.) (12)Australian (958,959) (Zhao et al.) (19)Chinese (76,87) (Lu et al.) (14)Iranian (65, 65) (Abutorabi et al.) (16)Iranian (150, 150) (This study)^**^
-238G/A	Iranian (150, 150) (This study)£	Korean (70, 202) (Lee et al.) (17)Austrian (92, 69) (Wieser et al.) (18)Japanese (130, 185) (Asghar et al.) (12)Australian (958, 959) (Zhao et al.) (19)Iranian (65, 65) (Abutorabi et al.) (16)Iranian (149, 150) (This study)^**^


BMI; Body mass index, ^*^; Allele frequencies, ^**^; Genotype adjusted by age and BMI, and ^£^; case and control and BMI adjusted by age.

## Discussion

Endometriosis is a multifactorial disease with both genetic and environmental components (8). Studies have scanned the genome and specific candidate genes to determine the genetic aetiology of this disease (22), reporting on endometriosis and related genes involved in "detoxification, galactose metabolism, steroid hormone production and inflammation" (15, 21, 22). Studies have also shown that any change in function and number of immune cells as well as high levels of inflammatory cytokines may lead to endometriosis (23). The present study aimed at examining the association of four *TNF-α* polymorphisms, -238G/A, -308G/A, -857C/T and -863C/A with endometriosis in an Iranian sample.

The effects of polymorphisms on cytokines genes have been examined by many studies (22) as well as the possible association of *TNF-α* polymorphisms with augmented endometriosis risk (12, 13, 15, 17-20). So far, many polymorphic variants have been examined in different populations, leading to various results (24). A number of polymorphisms have been associated with the disease in many populations, however, in some studies no association with the disease was observed. The reason for these observed differences may include different diagnostic criteria in selection of patients and controls, distinct living setting, number of samples, and varying populations and ethnicities (24, 25).

All in all, endometriosis has been reported to be linked with a number of polymorphisms of *TNF-α*. The study by Asghar et al. (12) and two other investigations by Lee et al. (17) concluded that -238G/A, -308G/A, -857C/T and -863C/A polymorphisms in the promoter region of *TNF-α* had no impact on women developing endometriosis. In contrast, these studies and that by Saliminejad et al. (15) showed that the frequency of the -1031C allele in the *TNF-α* gene in patients suffering severe endometriosis was significantly lower than that of their control group. Additional genetic studies on promoter polymorphisms in *TNF-α* by Wieser et al. (18) (238 G/A and-308G/A), and Hsieh et al. (13) and Lu et al. (14) (308G/A) found no association with endometriosis in the Asian population. Another study by Zhao et al. (19) in Australia reported that the -863C/A polymorphisms in *TNF-α* had no effect on patients with endometriosis. Moreover, Abutorabi et al. (16) found a positive association between the -1031 T/C polymorphism with endometriosis. However, no significant association was observed between the -238 G/A and -308 G/A polymorphisms with the disease. A similar study by Teramoto et al. (20) discovered an over- representation between the TNF-U01 haplotype (1031T-863C and, 857C) and endometriosis in Japanese women.

According to the studies mentioned, *TNF-α* -238G>A has been inspected in three studies, -308G>A in five articles, -857C>T in three articles and -863C>A in four articles, all of which showed compatible findings where no significant association was reported between these four polymorphisms and endometriosis in any of the models. Our study reported an association between the -863C/A polymorphism in the promoter region of *TNF-α* and endometriosis. We also observed a direct relationship between the case and control and BMI when adjusting by age (Table 3).

It has been shown that the presence of polymorphism in promoter regions can affect gene expression (26). STRING showed that *TNF-α* interacts with 10 other molecules. These interactions are likely to be functionally important, especially those with proteins involved in cell survival and apoptosis such as TRAF2, which regulates activation of *NF-Kappa-B* and *JNK* and has a central role in the regulation of cell survival and apoptosis (27, 28). Also, the interaction with *TNFRSF1B* (receptor with high dependency for *TNFSF2/TNF-α*) is essential for mediating most of the metabolic efficacy of *TNF-α* (27, 29).

This study has some limitations in spite of its strengths. The limitations of our study on endometriosis were not only the difficulty in choosing the controls, but also in recruiting patients. This is because laparoscopy should be undertaken to confirm the disease and its steady state that was a matter of time to collect samples.

## Conclusion

We investigated the association of four polymorphisms in the promoter region of *TNF-α* in Iranian women with endometriosis (stages I-IV of disease). *TNF-α* -863 A allele was significantly lower in women with endometriosis than controls, suggesting that the -863 A allele may play a role in incidence of endometriosis among Iranian women. Development of endometriosis among those people with -863 A allele seems low although it should be noted that the calculations show is not significant when adjusting for multiple testing. According to the results, the current study indicates that there might be a direct relationship between BMI and progression of endometriosis.
